# Comparison of repeated surgical resection and radiofrequency ablation for small recurrent hepatocellular carcinoma after primary resection

**DOI:** 10.18632/oncotarget.21604

**Published:** 2017-10-07

**Authors:** Wei-Chi Sun, I-Shu Chen, Huei-Lung Liang, Cheng-Chung Tsai, Yu-Chia Chen, Being-Whey Wang, Huey-Shyan Lin, Hoi-Hung Chan, Ping-I Hsu, Wei-Lun Tsai, Jin-Shiung Cheng

**Affiliations:** ^1^ Division of Gastroenterology and Hepatology, Department of Internal Medicine, Kaohsiung Veterans General Hospital, Kaohsiung, Taiwan; ^2^ Shool of Medicine, National Yang-Ming University, Taipei, Taiwan; ^3^ Division of General Surgery, Department of Surgery, Kaohsiung Veterans General Hospital, Kaohsiung, Taiwan; ^4^ Department of Radiology, Kaohsiung Veterans General Hospital, Kaohsiung, Taiwan; ^5^ School of Nursing, Fooying University, Kaohsiung, Taiwan

**Keywords:** hepatocellular carcinoma, radiofrequency ablation, resection

## Abstract

There is controversy concerning whether radiofrequency ablation (RFA) or surgical resection (SR) is a better treatment option for recurrent HCC after resection. In Kaohsiung Veteran General Hospital, from January 2002 to September 2014, a total of 100 consecutive patients who developed recurrent HCCs with a tumor size ≦ 3 cm and tumor numbers ≦ 3 after surgical resection were enrolled. Among these patients, 57 patients received RFA and 43 patients underwent repeated SR. Baseline characteristics at the time of recurrence after hepatic resection and clinical outcomes following treatment of recurrent HCC were compared between the two groups. The baseline data of initial HCC and the first recurrence of HCC were comparable in both groups. The 1-, 3-, 5-year overall survival rates following treatment of the first recurrence of HCC were 97.6%, 82.7%, 56.4% in the repeated SR group and 98.2%, 77.2%, 52.6% in the RFA group (*p* = 0.69). The 1-, 3-, 5-year disease-free survival rates were 57.0%, 32.1%, 28.6% in the repeated SR group and 60.8%, 26.6%, 16.6% in the RFA group ((*p* = 0.89). There was a trend whereby patients who underwent repeated SR had more procedure-related morbidity than patients who underwent RFA (16% vs. 7%, *p* = 0.14). The median total hospital days were longer in the repeated SR group than that in the RFA group (13 vs. 5 days, *p* < 0.05). In the small recurrent HCCs after SR, RFA achieved similar overall survival and disease-free survival than those with repeated SR as well as having a shorter hospital stay.

## INTRODUCTION

The curative treatment modalities for hepatocellular carcinoma (HCC) include liver transplantation, surgical resection (SR), and radiofrequency ablation (RFA). Although liver transplantation is the best treatment option for patients with HCC, SR and RFA are most commonly considered first-line treatments because of the graft shortage. Numerous studies suggest both SR and RFA are comparable in terms of long-term survival for patients with early stage HCC. The 5-year survival rates are 42%–56% for SR and 42%–70% for RFA [1–6]. However, even in patients who underwent liver transplantation, tumor recurrence is not uncommon with a 10%–15% risk of recurrence [7]. The 5-year recurrence rate after SR is 42%–52% [8–11], which is higher up to 42%–70% after RFA [12, 13]. Therefore, how to manage recurrent HCC is important in improving the survival and merits further evaluation. Of all the recurrence patterns, intrahepatic recurrence is the most common and the size of the recurring HCC is usually smaller than that of the initial HCC because the surveillance interval is shorter [14]. Accordingly, the treatment options do not particularly differ between recurrent HCC after SR and primary tumor. Liver transplantation is still recognized as the better choice for recurrent HCC [15], but its wide application has been limited by the shortage of donors. Repeated SR for recurrent HCC has been reported to be an effective treatment option with a comparable survival rate to that of primary SR [16–18], but its feasibility is limited by small liver remnants, poor liver function reserve, or technical difficulties owing to expected postoperative adhesion [19–21]. RFA, as a nonsurgical, less invasive, and repeatable therapeutic approach, is safer and causes less damage in treating recurrent HCC following primary resection [22–24]. As to the choice of repeated SR and RFA, although several studies have compared the clinical outcome of RFA vs. SR for recurrent HCC, no clear recommendation has yet been established. Many of the studies were limited by small case numbers or large tumor size [25–31] and there remain many controversies regarding the choice of repeated SR and RFA in treating recurrent tumor after primary resection. This study aimed to compare the efficacy, safety, and long-term survival of repeated SR and RFA for recurrent HCCs.

## MATERIALS AND METHODS

This study was conducted as a retrospective chart review and analysis of a prospective database from the cancer center in our hospital. Our study was approved by the institutional review board of our hospital and informed consent was waived.

### Patients

**Figure 1:**
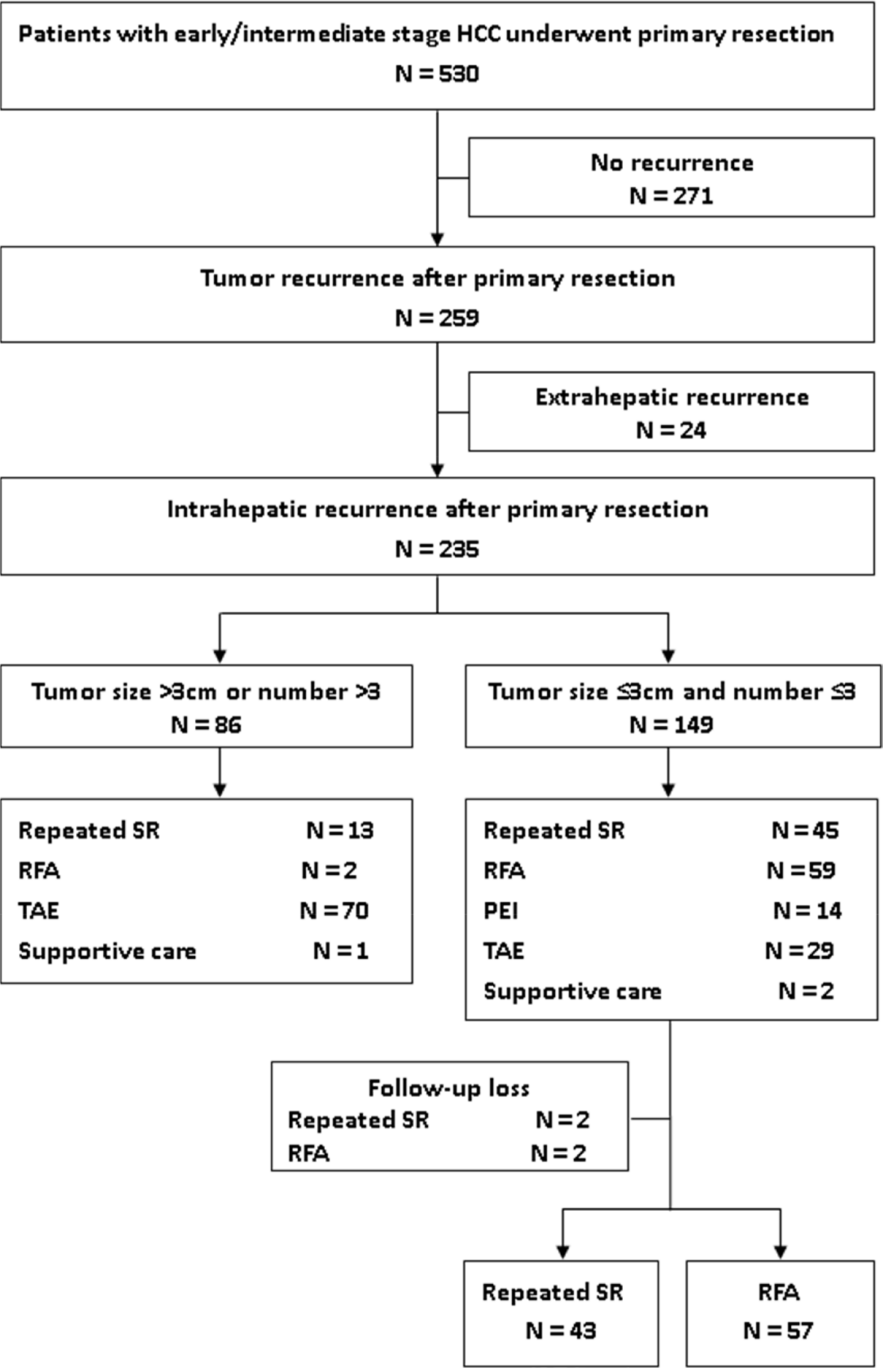
Flowchart summarizes patient inclusion PEI = percutaneous ethanol injection, RFA = radiofrequency ablation, SR = surgical resection, TAE = transcatheter arterial embolization.

From January 2002 to September 2014, a total of 530 consecutive patients with early and intermediate stage HCC underwent hepatic resection at the Division of General Surgery, Department of Surgery, Kaohsiung Veteran General Hospital. At the time of the data collection and analysis, 271 patients were free of tumor recurrence, 235 patients had intrahepatic recurrence, and 24 patients had extrahepatic recurrence. For matching comparison between repeated surgical resection and RFA, patients with fewer or equal to 3 recurrent tumors and each tumor size less than or equal to 3cm were enrolled. The detailed inclusion and exclusion of patients were shown in Figure 1. Among the 235 patients with intrahepatic recurrence, 100 patients with fewer or equal to 3 recurrent tumors and each tumor size ≦ 3cm received either repeated SR or RFA as the secondary treatment, and these patients were enrolled in our study The data of patient characteristics, clinicopathologic features, and survival outcomes were reviewed.

### Surveillance and diagnosis for recurrent HCC

In our hospital, all patients received computed tomography (CT) or magnetic resonance imaging (MRI) of the liver within one to two months after primary hepatectomy of the HCC to confirm complete tumor clearance. Thereafter, surveillance for recurrent HCC consisted of measurements of serum alpha-fetoprotein (AFP), liver biochemistry, and ultrasonography, CT scan, or MRI scans of the liver every three months. Intrahepatic recurrence was defined as a new lesion with arterial contrast enhancement and portal venous washout. If there was a new lesion less than 1cm and without typical HCC imaging pattern on contrast-enhanced CT or MRI scans, we arranged image studies every three months. If there was a new lesion more than 1cm and without a typical HCC imaging pattern on contrast-enhanced CT or MRI scans, we would arrange a needle biopsy for histological confirmation.

### Treatment strategy selection for recurrent HCC

Neither SR and RFA were adopted for recurrent HCC treatment if a patient had any symptoms and signs of irreversible liver decompensation and severe portal hypertension such as jaundice, ascites, encephalopathy, PTINR >1.5x, the presence of severe varix, and thrombocytopenia with a platelet count < 50 x× 10^9^/Cumm. Repeated hepatic resection was considered if a patient had a single tumor or tumors within a monosegment of liver with good liver function reserve. Repeated hepatic resection was generally avoided if patients had gross ascites, an indocyanine green (ICG) retention rate of more than 20% at 15 minutes and/or a serum total bilirubin level of more than 1.5 mg/dL, or the presence of moderate esophageal varix. RFA was generally selected in patients with Child-Pugh class A or B disease, prothrombin time ratio of more than 50%, and platelet count of more than 50 000/mm^3^ (50 × 10^9^/L). RFA also was considered if the indocyanine green (ICG) retention rate was more than 20% at 15 minutes or recurrent tumors in a deep-seated intraparenchymal location where anatomical resection would remove more than one segment of liver, resulting in insufficient liver reservation. CT guided RFA was more preferred than sono-guided RFA if the tumor size was small and invisible by sonogram, difficult to approach due to altered anatomy, and tumors in high-risk locations, which was defined as tumors less than 5 mm adjacent to the hollow viscera, big bile duct, gallbladder, diaphragm, liver capsule, liver hilum, heart, major portal or hepatic vein.

### Statistical analysis

Continuous variables were expressed as medians and interquartile ranges and were compared using the Mann–Whitney *U*-test. Categorical variables were compared with the χ^2^ test or Fisher’s exact test when appropriate. Overall survival, disease-free survival, and cumulative incidence of second recurrence of the two study groups were estimated by the Kaplan-Meier method. Comparison of survival between groups was performed with the log-rank test. Disease-free survival following treatment of the first recurrence of HCC was defined as the period from the date of treatment of the first recurrence of HCC to the date of the second tumor recurrence or death. Overall survival after treatment of the first recurrence of HCC was defined as the period from the date of treatment of the first recurrence to the date of death related to any cause. Univariate and multivariate analyses of prognostic factors with the overall survival were evaluated by step wise forwards Cox’s regression analysis. A *p* value ≤ 0.05 was considered significant. Statistical analysis was performed using the SPSS 19.0 computer software program.

## RESULTS

### Baseline profile of primary HCC

Table 1 shows the patient and tumor characteristics of the initial HCC. The age, gender ratio, incidence of comorbidity, and positive rates of viral hepatitis, and laboratory data were comparable in both groups. In the repeated SR group, 35 (81.4%) patients had Child-Pugh class A cirrhosis and 1 (2.3%) had Child-Pugh class B cirrhosis, whereas 50 (87.7%) patients in the RFA group had Child-Pugh class A cirrhosis (*p* = 0.41). In each group, 7 patients did not have cirrhosis, which was based on radiological finding and Ishak score of non-tumor part in the surgical pathology. The two groups had similar clinico-pathological features of initial HCC in terms of tumor size, tumor number, distribution of tumor location, proportion of tumor BCLC staging, histological grade of tumor cell differentiation, and presence of microvascular invasion. Most patients had laparotomy segmentectomy or wedge resection for treatment of the initial HCC, and only four (7%) patients in the RFA group received a lobectomy.

**Table 1:** Patient and tumor characteristics of initial HCC

	Repeat SR (*n* = 43)	RFA (*n* = 57)	*P*
Age (years)	60 (35–76)	63 (27–81)	0.28
Gender M/F	34 (79%)/9 (21%)	38 (67%)/19 (33%)	0.19
Comorbidity (DM, CHF, COPD, CKD)	14 (33%)	19 (33%)	1.00
Risk factor of HCC			
viral hepatitis B	21 (49%)	32 (56%)	0.55
viral hepatitis C	20 (47%)	23 (40%)	0.55
alcohol	2 (5%)	4 (7%)	0.62
Cirrhosis severity			
Child-Pugh class A	35 (81%)	50 (88%)	0.41
Child-Pugh class B	1 (2%)	0 (0%)	0.25
Laboratory data			
serum bilirubin (mg/dL)	0.7 (0.3–1.4)	0.7 (0.2–2.3)	0.26
serum alanine transaminase (U/L)	92 (1–1808)	58 (1–221)	0.42
serum aspartate transaminase (U/L)	106 (1–1724)	72 (17–284)	0.40
serum albumin (g/dL)	3.9 (2.7–4.7)	3.9 (2.7–4.9)	0.60
platelet count (x103/Cumm)	169 (62–362)	177 (60–502)	0.62
PTINR	1.10 (0.97–1.83)	1.05 (0.91–1.31)	0.06
serum creatinine (mg/dL)	1.6 (0.7–12.1)	1.5 (0.6–12.8)	0.92
serum a-fetoprotein (ng/mL)	602 (1–11681)	1090 (3–29141)	0.54
ICG (%)	12.3 (2.6–28.0)	14.4 (0.8–48)	0.26
Primary tumor stage			
BCLC stage 0	5 (12%)	8 (14%)	0.77
BCLC stage A	29 (67%)	34 (60%)	0.42
BCLC stage B	9 (21%)	15 (26%)	0.53
Initial tumor size (cm)	3.9 (1.0–16.0)	3.9 (1.3–15.0)	0.94
Initial tumor number	1.1 (1–4)	1.2 (1–3)	0.60
Initial tumor number 1/> 1	36 (84%)/7 (16%)	48 (84%)/9 (16%)	0.58
Initial tumor location			
monosegment/disegment	41 (95%)/2 (5%)	55 (96%)/2 (4%)	0.77
Surgery method			
laparotomy/laparoscopy	39 (91%)/4 (9%)	55 (97%)/2 (3%)	0.23
lobectomy/segmentectomy/wedge resection	0(0%)/42 (98%)/1 (2%)	4(7%)/40 (70%)/13 (23%)	0.11
Histologic grade of initial tumor			
grade I	0 (0%)	2 (4%)	0.22
grade II	23 (54%)	30 (53%)	0.55
grade III	19 (44%)	24 (42%)	0.84
grade IV	1 (2%)	1 (1%)	0.84
Microvascular invasion	8 (19%)	7 (12%)	0.41

### Baseline profile of recurrent HCC

With the regards to the recurrent HCC (Table 2), the median time from initial resection to first recurrence was longer in the repeated SR group, but the there was no statistically significant difference (26 vs. 14 months, *p* = 0.06). The clinical characteristics of patients and the recurrent tumor were not significantly different in the two groups including laboratory data, tumor size, tumor number, and tumor staging. In the repeated SR group, 41 (90.7%) patients underwent segmentectomy and four (9.3%) wedge resections. In the RFA group, most (81%) procedures were performed via a CT guided approach, and 46 (81%) patients received one session of RFA, whereas 11 (19%) needed two sessions of RFA for complete treatment of recurrent tumors.

**Table 2:** Patient and tumor characteristics of first recurrent HCC

	Repeat SR (*n* = 43)	RFA (*n* = 57)	*P*
Median time from initial resection to 1st recurrence (m)	26 (4–126)	14 (1–86)	0.06
Age (years)	63 (37–84)	65 (31–84)	0.51
Cirrhosis severity			
Child-Pugh class A	35 (81%)	50 (88%)	0.41
Child-Pugh class B	1 (2%)	0 (0%)	0.25
Laboratory data			
serum bilirubin (mg/dL)	0.6 (0.1–1.3)	0.7 (0.3–1.8)	0.09
serum alanine transaminase (U/L)	39 (1–131)	49 (1–205)	0.06
serum aspartate transaminase (U/L)	48 (1–162)	51 (1–206)	0.62
serum albumin (g/dL)	3.9 (2.7–4.6)	3.9 (3.0–4.8)	0.85
platelet count (x103/Cumm)	165 (90–321)	149 (78–305)	0.12
PTINR	1.03 (0.91–1.21)	1.08 (0.89–2.12)	0.13
serum creatinine (mg/dL)	1.9 (0.7–13.1)	1.5 (0.1–10.4)	0.37
serum a-fetoprotein (ng/mL)	23 (3–290)	67 (2–817)	0.19
ICG (%)	14.0 (4.0–34.0)	22.0 (19.0–25.0)	0.17
Recurrent tumor stage			
BCLC stage 0	17 (40%)	32 (56%)	0.11
BCLC stage A	26 (60%)	25 (44%)	0.11
Recurrent tumor size (cm)	1.9 (0.8–3.0)	1.8 (1.0–3.0)	0.17
Recurrent tumor number	1.2 (1–3)	1.1 (1–2)	0.14
Recurrent tumor number 1/> 1	41 (95%)/2 (5%)	55 (96%)/2 (4%)	0.77
Recurrent tumor location			
monosegment/disegment	41 (95%)/2 (5%)	55 (96%)/2 (4%)	0.77
Repeat surgery method			
lobectomy/segmentectomy/wedge resection	0 (0%)/39 (91%)/4 (9%)	-	-
Radiofrequency ablation			
CT/ultrasound guided method	-	46 (81%)/11(19%)	-
Total RFA session	-	1.1 (1–2)	-
RFA session 1/2	-	53 (93%)/4 (7%)	-

### Clinical outcome after treatment of recurrent HCC

Table 3 showed the postoperative and long term outcomes of patients receiving repeated SR and RFA for the treatment of the first recurrence of HCC. Seven (16%) patients developed a total of 10 operation related complications in the repeated SR group, and one (2%) patient experienced a major complication: death due to bile duct injury with bile leakage and septic shock. In the RFA group, four (7%) patients developed a total of six procedure related complications, and there was no complication related mortality. The incidences of treatment related morbidity (*p* = 0.14) and mortality (*p* = 0.25) did not differ in the two groups. The total hospital day was longer in the repeated SR group (13 vs. 5 days, *p* < 0.05). The overall treatment response rates were similar (repeated SR 100% vs. RFA 98%, *p* = 0.67). At the time of analysis, 30.2% of patients over the median follow up time of 53 months in the repeated SR group and 28.1% patients over the median follow up time of 54 months in the RFA group remained free of a second recurrence. The median time from the treatment of recurrent HCC to developing a second recurrence was 11 months in the repeated SR group and 10 months in the RFA group (*p* = 0.74). Intrahepatic recurrence was the most common recurrence pattern in both groups. For the treatment of the second recurrence of HCC, 55% of patients received curative treatment in the repeated SR group, whereas 67% of patients received curative treatment in the RFA group.

**Table 3:** Postoperative and long term outcomes after treatment of first recurrent HCC

	Repeat SR (*n* = 43)	RFA (*n* = 57)	*P*
Operation related complications	7 (16%)	4 (7%)	0.14
Pleural effusion	1	2	0.73
Pneumothorax	0	1	0.38
Wound infection	1	0	0.25
Intraabdominal hemorrhage	0	0	1.00
Sepsis	5	2	0.12
Bile duct injury/Bile leakage	3	1	0.19
Hospital mortality	1 (2%)	0 (0%)	0.25
Total hospital day (day)	13 (5–27)	5 (2–17)	< 0.05
Overall treatment response	100%	98%	0.67
Complete response	42	55	
Partial response	1	1	
Stable disease	0	1	
Progressive disease	0	0	
Median time from 1st recurrence to 2nd recurrence (m)	11 (4–82)	10 (0–87)	0.74
2nd recurrence pattern			
No recurrence	13 (30%)	16 (28%)	
Intrahepatic recurrence	29 (67%)	39 (68%)	
Extrahepatic recurrence	1 (3%)	2 (4%)	
Management of second intrahepatic recurrence			
OP	8 (27%)	3 (8%)	
PEI	2 (7%)	7 (18%)	
RFA	6 (21%)	16 (41%)	
TACE	13 (45%)	13 (33%)	
Others (HAIC, Sorafenib, R/T, palliation)	0 (0%)	0 (0%)	

### Comparison of survival results between the groups

**Figure 2:**
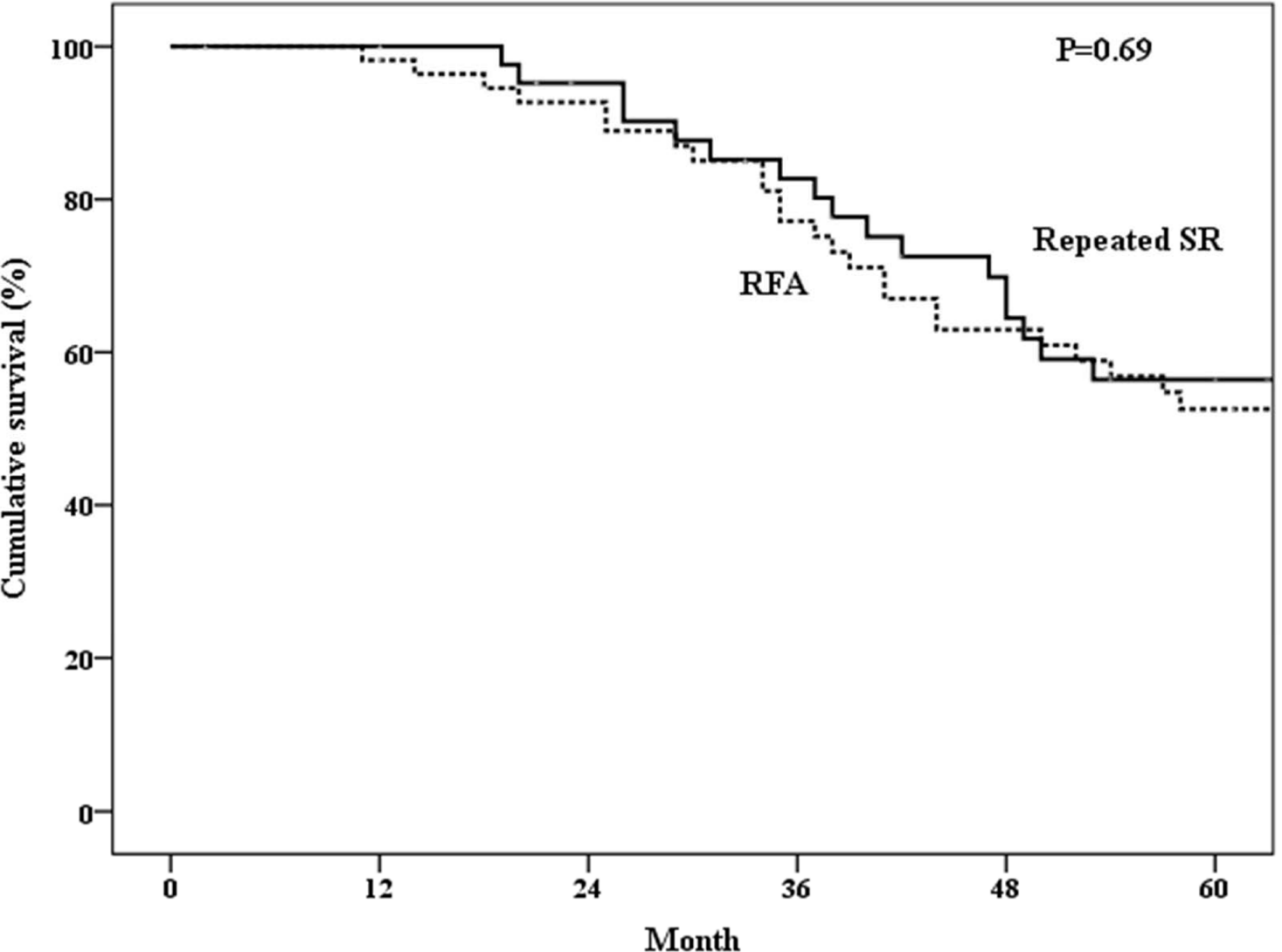
Overall survival of patients who underwent repeated surgical resection (SR) or radiofrequency ablation (RFA)

**Figure 3:**
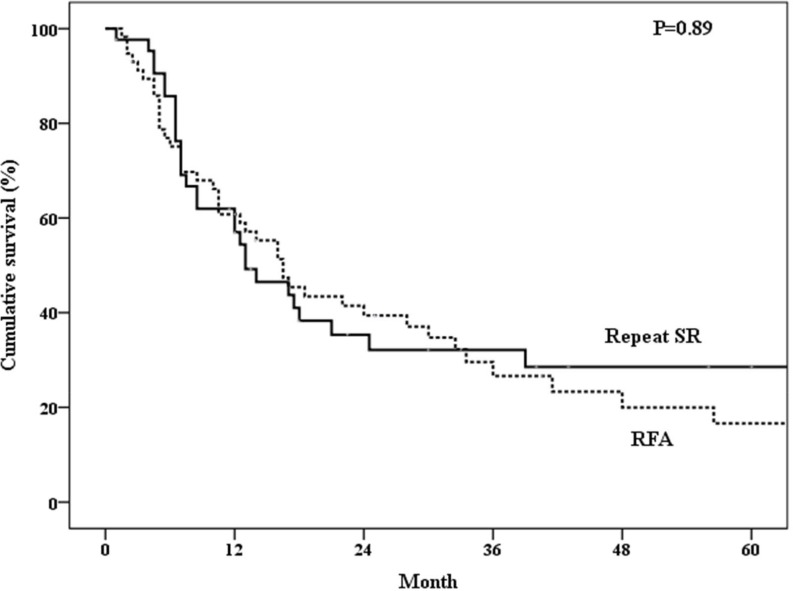
DDisease free survival of patients who underwent repeated surgical resection (SR) or radiofrequency ablation (RFA)

**Figure 4:**
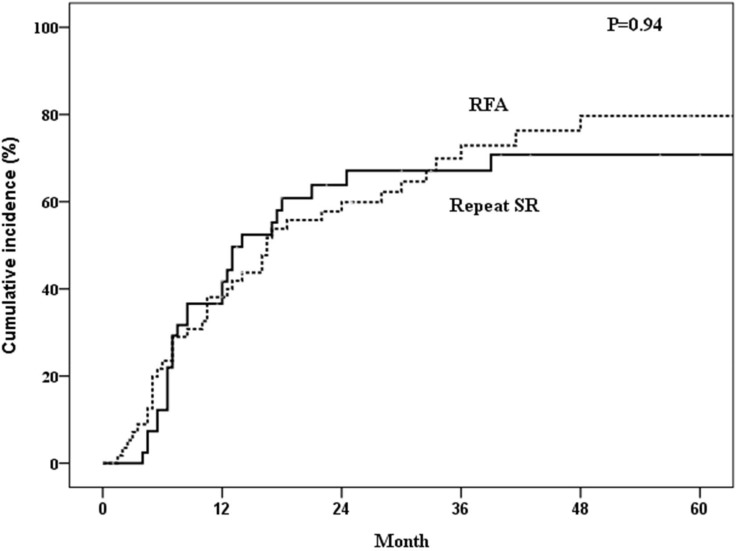
Incidence of second recurrence of patients who underwent repeated surgical resection (SR) or radiofrequency ablation (RFA)

The 1-, 3-, 5-year overall survival rates after treatment of the first recurrence of HCC were 97.6%, 82.7%, 56.4% in the repeated SR group and 98.2%, 77.2%, 52.6% in the RFA group (*P* = 0.69) (Figure 2). The 1-, 3-, 5-year disease-free survival rates were 57.0%, 32.1%, 28.6% in the repeated SR group and 60.8%, 26.6%, 16.6% in the RFA group (*P* = 0.89) (Figure 3). The 1-, 3-, 5-year cumulative incidences of the second recurrence after treatment of the first recurrence of HCC were 41.7%, 67.1%, 70.8% in the repeated SR and 28.1%, 72.9%, 79.7% in the RFA group (*P* = 0.94) (Figure 4). In the Cox’s regression analysis, with regard to overall survival, the histologic grade of the initial tumor (I vs non-I), AFP level at the time of tumor recurrence, time from initial resection to 1^st^ recurrence, and time from treatment of 1^st^ recurrent HCC to 2^nd^ recurrence were significant prognostic factors at univariate analysis; HBsAg positive, age at the time of tumor recurrence, the histologic grade of the initial tumor (I vs non-I), AFP level at the time of tumor recurrence, time from initial resection to 1^st^ recurrence, and time from treatment of 1^st^ recurrent HCC to 2^nd^ recurrence were significant prognostic factors at multivariate analysis (Table 4).

**Table 4:** Univariate and multivariate analyses of prognostic factors for overall survival

	Univariate Analysis			Multivariate Analysis		
	HR	95% CI	*P*	HR	95% CI	*P*
RFA & Repeated SR	1.136	0.610–2.116	0.687	1.058	0.375–2.988	0.915
Gender	1.868	0.997–3.501	0.051	0.405	0.088–1.872	0.247
HBsAg positive	0.860	0.464–1.594	0.631	0.136	0.026–0.720	0.019
Anti-HCV-Ab positive	0.916	0.496–1.689	0.778	0.498	0.109–2.275	0.368
Liver cirrhosis	1.171	0.559–2.453	0.676	6.172	0.940–40.50	0.058
Histologic grade of initial tumor (I vs non-I)	10.04	2.209–45.64	0.003	242.4	10.12–5800.7	0.001
Microvascular invasion of initial tumor	0.842	0.353–2.005	0.697	1.572	0.399–6.193	0.518
Age at the time of tumor recurrence	1.011	0.982–1.041	0.465	1.075	1.012–1.143	0.019
Recurrent tumor size	1.002	0.604–1.662	0.994	2.532	0.932–6.875	0.068
Recurrent tumor number	1.423	0.741–2.733	0.290	0.700	0.172–2.856	0.620
AFP level at the time of tumor recurrence	1.004	1.001–1.007	0.007	1.005	1.001–1.009	0.014
Time from initial resection to 1st recurrence	0.974	0.957–0.991	0.003	0.965	0.940–0.991	0.009
Time from treatment of 1st recurrent HCC to 2nd recurrence	0.973	0.954–0.993	0.008	0.949	0.911 – 0.989	0.013

## DISCUSSION

Our study suggested RFA achieves long-term survival outcomes similar to repeated SR in small recurring HCCs less than 3cm after primary resection. Previous studies comparing the efficacy of repeated RFA vs. resection after recurrence of HCCs usually also enrolled recurring tumors of more than 3cm in size (25, 28, 30, 31). Actually, following surgical resection of the primary tumors, intensive screening was usually applied and the recurring tumors were usually smaller than 3cm and the recurrent HCCs in our study were relatively small: 46% were smaller than 2cm, which was more like the real situation in the recurrence of tumors. A recent meta-analysis [32] reported RFA was associated with lower disease-free survival rates, however, our study we found both SR and RFA achieved a similar outcome for recurrent HCCs.. This might be explained by the inclusion criteria whereby the recurrent HCCs in our study were relatively small. As expected, a smaller tumor size is closely related to a higher rate of complete tumor elimination after RFA and a greater safety margin with fewer non-tumorous liver parenchyma resections in repeated SR, so the outcomes will be better. Besides, most RFA were performed under sonogram guidance in previous reports, but most (81%) patients received RFA under CT guidance in our study because the tumor size was usually small and some were not visible by sonogram. Altered anatomy after resection also contributed to difficulty with US guided RFA. Some experts suggested CT-guided RFA provides better detection of RFA lesions, margin discrimination, immediate ablation zone evaluation, and few artifacts [33], which may achieve better complete tumor ablation and better outcomes. Indeed, the RFA treated group in our study had a better 5-year overall survival rate and disease free survival rates than several previous studies [25–28]. Although in a recent study, Lee et al. found that either US or CT guided RFA was comparable for treatment naïve HCC [34], there was no study that compared the efficacy and safety of US or CT in the guidance of RFA for recurrent HCC after primary resection. This interesting problem merits further investigation in the future.

Previous studies found that patients with a tumor size ≦ 3cm and tumor numbers ≦ 3 benefited most from RFA treatment, so this is the reason we included only patients with tumor size ≦ 3cm and tumor numbers ≦ 3.

In our study, both repeated SR and RFA completely eliminated recurrent HCC and achieved near 100% response rates. However, a second recurrence of HCC is not uncommon. Chan et al [28] showed 72.4% of patients in the repeated SR group and 84.4% of patients in the RFA group developed a second recurrence with a similar median time from treatment of the first recurrence to second recurrence (6.3 vs. 9.5 months, *p* = 0.25). Our study showed 79.8% of patients in the repeated SR group and 71.9% of patients in the RFA group developed a second recurrence with a similar median time from treatment of the first recurrence to second recurrence (11 vs. 10 months, *p* = 0.74).

The improvement in liver function evaluation, surgical technique and perioperative care and decrease in postoperative morbidity and mortality make it possible for more patients to receive surgical resection [16, 35, 36]. However, surgical resection of recurrent HCC is still challenging due to small liver remnants, poor liver function reserve, intra-abdominal adhesion from previous surgery, and the tumor location being adjacent to major vascular or biliary structures. Although the latest literatures suggest repeated SR is considered a favorable and important curative treatment for recurrent HCC, postoperative complications, and in particular hepatic failure, are not uncommon and can not be overlooked [37]. A meta-analysis study showed repeated SR for recurrent HCC was associated with a higher procedure related morbidity rate compared with RFA [32]. Our study showed the procedure-related complication rates were higher in the repeated SR group than that in the RFA group (16% vs. 7% *p* = 0.14). One patient with repeated SR had major complications and died of bile duct injury with bile leakage and septic shock. RFA is a minimally invasive procedure and can be performed percutaneously either by ultrasound or CT guided approach. Compared with repeated SR via the open or laparoscopic approach, RFA is a highly target-selective thermal treatment technique to conserve non-tumorous liver parenchyma and minimize the degree of surgical insult to the limited liver reserve. Apart from surgery, RFA can be performed under conscious sedation and has a shorter hospital day, making it more cost-effective than surgical resection [2, 38]. In our study, median total hospital stay for patients who underwent RFA was significantly shorter than for those who underwent repeated SR (5 vs. 13 days, *p* < 0.05). Further, the characteristics and benefits of less invasiveness and highly-targeted tumor treatment improved the feasibility of patients and repeatability of RFA for recurrent HCC. Our study showed more than one third of patients in both groups underwent loco-regional treatment for a second recurrence of HCC, whereas 8% of patients in the RFA group and 27% of patients in the repeated SR group were amenable to surgical resection of a second recurrence of HCC. The aforementioned beneficial factors of RFA make it more safe and feasible in treating recurrent HCC after hepatectomy.

Previous studies reported the serum albumin level, serum alpha fetoprotein level, recurrent tumor size, time interval from primary hepatectomy to first recurrence, and time interval from treatment of recurrent HCC to second recurrence were significant prognostic factors to overall survival [23, 25, 28]. A shorter time interval from treatment to recurrence, is associated with poor prognosis. The finding of our study is consistent with the result of previous report by Albert C.Y. Chan, et al in 2012 [39]. The univariate and multivariate analyses in our study also showed similar findings, except recurrent tumor size was not related to overall survival. Our finding can be explained by the relatively small recurrent tumor size in our study and complete elimination rate of recurrent tumor increase with a decreased possibility of the presence of satellite nodules which decrease early recurrence and improve overall survival. Moreover, the presence of HBV infection and younger age at the time of tumor recurrence were significant factors of better overall survival at univariate analysis. Although an acceptable long term survival is reachable in carefully selected elderly patients [40, 41], patient age is theoretically related to the post-treatment complication and disease morbidity and is always more relevant for the prognosis than many tumor and treatment factors. However, the reason why the presence of HBV infection was also a favorable factor was unknown. It was probably because all HBV patients with indication (45.3%) in our study had anti-viral agent treatment, which had been proved to reduce HBV related long-term complications and lead to better prognosis [42].

The limitation of our study included non-randomization. Although this was not a randomized study, the baseline characteristics including the ICG retention rate at 15 minutes at initial diagnosis of HCC and at recurrence of HCC after surgical resection between the two groups of patients were similar. A prospective randomized controlled trial to compare the efficacy or repeated RFA and surgical resection for recurrent HCCs after surgical resection is required.

## CONCLUSIONS

In small recurrent HCCs after SR, RFA achieved similar overall survival and disease-free survival than those with repeated SR and also had a shorter hospital stay.
